# Solar enhanced oxygen evolution reaction with transition metal telluride

**DOI:** 10.3389/fchem.2024.1381144

**Published:** 2024-04-26

**Authors:** Harish Singh, Taishi Higuchi-Roos, Fabrice Roncoroni, David Prendergast, Manashi Nath

**Affiliations:** ^1^ Department of Chemistry, Missouri University of Science and Technology, Rolla, MO, United States; ^2^ Department of Chemical and Biochemical Engineering, Missouri University of Science and Technology, Rolla, MO, United States; ^3^ Joint Center for Energy Storage Research, the Molecular Foundry, Lawrence Berkeley National Laboratory, Berkeley, CA, United States

**Keywords:** photo-coupled electrochemical oxygen evolution reaction, nickel telluride, density functional theory (DFT, ), solar water splitting, solar hydrogen

## Abstract

The photo-enhanced electrocatalytic method of oxygen evolution reaction (OER) shows promise for enhancing the effectiveness of clear energy generation through water splitting by using renewable and sustainable source of energy. However, despite benefits of photoelectrocatalytic (PEC) water splitting, its uses are constrained by its low efficiency as a result of charge carrier recombination, a large overpotential, and sluggish reaction kinetics. Here, we illustrate that Nickel telluride (NiTe) synthesized by hydrothermal methods can function as an extremely effective photo-coupled electrochemical oxygen evolution reaction (POER) catalyst. In this study, NiTe was synthesized by hydrothermal method at 145°C within just an hour of reaction time. In dark conditions, the NiTe deposited on carbon cloth substrate shows a small oxygen evolution reaction overpotential (261 mV) at a current density of 10 mA cm^–2^, a reduced Tafel slope (65.4 mV dec^−1^), and negligible activity decay after 12 h of chronoamperometry. By virtue of its enhanced photo response, excellent light harvesting ability, and increased interfacial kinetics of charge separation, the NiTe electrode under simulated solar illumination displays exceptional photoelectrochemical performance exhibiting overpotential of 165 mV at current density of 10 mA cm^-2^, which is about 96 mV less than on dark conditions. In addition, Density Functional Theory investigations have been carried out on the NiTe surface, the results of which demonstrated a greater adsorption energy for intermediate -OH on the catalyst site. Since the -OH adsorption on the catalyst site correlates to catalyst activation, it indicates the facile electrocatalytic activity of NiTe owing to favorable catalyst activation. DFT calculations also revealed the facile charge density redistribution following intermediate -OH adsorption on the NiTe surface. This work demonstrates that arrays of NiTe elongated nanostructure are a promising option for both electrochemical and photoelectrocatalytic water oxidation and offers broad suggestions for developing effective PEC devices.

## 1 Introduction

The need for renewable and sustainable sources of energy is becoming increasingly evident as the world’s population continues to grow with increasing demand for energy and dwindling supply of fossil fuels. In recent years, hydrogen has been considered as the cleanest source of energy due to its high calorific value and lack of secondary pollution specifically, no green-house gas emission. However, the source for hydrogen generation can effectively categorize it as clean (green or yellow) or dirty hydrogen (grey or brown). Among these, hydrogen generated from electrocatalytic or solar-intensified water splitting (green and yellow hydrogen, respectively), have recently attracted significant interest due to their potential impact in advancing hydrogen economy. Electrochemical water splitting, typically includes the anodic oxygen evolution reaction (OER) and the cathodic hydrogen evolution reaction (HER) (Nath et al., 2022). However, the slow reaction kinetics along with high energy consumption and particularly the challenging OER process as the energy intensive step, have emerged as the major limitations for green/yellow hydrogen generation. This has led to significant efforts to design exceptionally efficient electrocatalysts to accelerate the sluggish kinetics of OER. Traditionally precious metal oxides like RuO_2_ and IrO_2_ have been used as conventional state-of-the-art electrocatalysts for OER, especially in acidic medium. However, their limited reserves and high cost, have prevented their widespread use in technologically relevant green hydrogen generating systems (Kong et al., 2012; Lee et al., 2016; Xu et al., 2016). Recent research efforts have witnessed the growth of transition metal based OER electrocatalysts with significantly higher electrocatalytic efficiency in alkaline medium, leading to potential application in fuel cells and water splitting devices (Wang et al., 2018; Li P. et al., 2021; Rana et al., 2021; Sanati et al., 2022; Sun et al., 2022). These transition metals based electrocatalysts, comprising mainly nickel, cobalt, and iron are appealing due to their accessibility, affordable price, and significantly enhanced OER activity in alkaline medium (Zhao et al., 2022). Among these, metal oxides, phosphides, sulfides, selenides, and tellurides have garnered substantial attention as possible electrocatalysts due to their tunable electrochemical activity, lattice stability and compositional variance (Menezes et al., 2017; Masud et al., 2018; Bhat and Nagaraja, 2019; Ghosh et al., 2019; Zhang et al., 2020; Li et al., 2021; Nath et al., 2021; Singh et al., 2022b; Singh et al., 2023). The transition metal oxides have shown good performance for OER electrocatalysis. However, their high bandgap and limited electrical conductivity along with higher overpotential for OER has limited their superiority compared to precious metal oxides (Rana et al., 2020; Gao et al., 2021; Zhang et al., 2021; Hu et al., 2022). The advancements made through doping and the use of carbon-based supports, significantly enhance the OER activity of transition metal oxides. The doping of specific heteroatoms, various structural refinements, and synthesis of many nanocomposites are the processes that have also been used to accelerate the oxygen evolution reaction (Umapathi et al., 2017; Guo et al., 2020). In comparison, transition metal selenides and tellurides have shown significantly better OER catalytic activity which has been primarily attributed to their higher charge transport, better electrochemical tunability of the catalytic site, and enhanced lattice covalency.

Recently, it has been shown that solar light-assisted electrocatalysis *via* the photo-electrocatalytic effect can be an easy and effective method for boosting electrocatalytic performance, serving as an extra push to reduce activation energy barriers and speed up the kinetics of electrochemical reactions (Shi et al., 2018; Gaikwad et al., 2022). Furthermore, integrating sustainable and renewable solar energy into the electrochemical process can not only assure the rational and full exploitation of resources, but also significantly boost the electrocatalyst’s activity. Using a light-driven carrier method, Min et al. proposed using defect-rich Fe-doped Co_3_O_4_, which showed minimal overpotential and remarkable endurance (Min et al., 2021). Furthermore, by employing a Ni-Fe-P-Ni_3_S_2_/NF heterogeneous electrocatalyst, Li et al. demonstrated that solar illumination may significantly boost the OER and HER properties (Li et al., 2021). Additionally, Zhang et al. demonstrated that the multifunctional Ni_3_S_2_ nanosheets could enhance the surface localized temperature through the *in situ* thermal effect in addition to producing photogenerated carriers, which facilitated OER performance (Zhang et al., 2020).

Due to its advantageous electrochemical activity in alkaline electrolyte solutions, nickel (Ni) has become the most widely used and widely available catalyst over the past few decades. Nickel exhibits various oxidation states (Ni^2+^/Ni^3+^) with the closest oxygen evolution potential to thermodynamic water splitting voltage (1.23 V vs RHE). Ni-based electrode materials, such as oxides, hydroxides, sulfides, selenides, and tellurides, are being employed extensively as prospective electrocatalysts for water splitting application, in comparison to noble metals like Pt, Ru, and Ir (Swesi et al., 2016; Zhang et al., 2020; Xue et al., 2020; Singh et al., 2022a; Gebreslase et al., 2022; Liu et al., 2022). Another factor that affect the electrocatalytic activity is the covalency in the metal-chalcogen bond and previous studies have demonstrated that increasing the degree of covalency in the metal-anion bonding improves OER catalytic efficiency. Since covalency increases as the electronegativity of the chalcogen atom decreases, it highlights the fact that catalytic efficiency will improve along the chalcogenide series from oxide to telluride (De Silva et al., 2018; Umapathi et al., 2020; Nath et al., 2021; Singh et al., 2021; 2022a; Saxena et al., 2022). In recent years, many Nickel-based selenide and telluride electrocatalysts have been described for water splitting, but few for high-efficiency solar-assisted electrocatalysis which could potentially offer the additional driving power needed to decrease the activation energy barriers. In this article, we have reported the growth of NiTe elongated nanostructure on carbon cloth through low temperature hydrothermal method which shows significantly enhanced OER activity under solar illumination. The NiTe electrode showed an overpotential of 165 mV under AM 1.5 solar illumination which depicted an improvement of 96 mV compared to OER activity in the absence of light. The NiTe elongated nanostructure were synthesized hydrothermally at low temperatures with a reaction time of an hour which makes this a very low energy expense rapid process that can be easily scaled up. Moreover, the light activated enhanced OER efficiency makes this NiTe composite as a promising candidate for photoelectrocatalytic water splitting with high intrinsic activity and low cost of operation.

## 2 Experimental section

### 2.1 Materials

All chemicals were used as is without further purification. Nickel sulfate (NiSO_4_·6H_2_O) was purchased from Alfa-Aeser, hydrazine hydrate (N_2_H_4_·H_2_O, 100%), isopropanol (IPA) and tellurium dioxide (TeO_2_) were purchased from Acros Organics. Carbon cloth (CC) substrate and Nafion were bought from Fuel Cells store and Ion Power, respectively. Before usage, the carbon cloth substrate was cleaned several times with acetone, ethanol, and distilled water.

### 2.2 Hydrothermal synthesis of nickel tellurides

First, 0.1 M TeO_2_ and 0.1 M NiSO_4_·6H_2_O were dissolved in 8 mL deionized water and stirred for 30 min. 3 mL of N_2_H_4_⋅H_2_O was then added to the above solution and stirred for another 20 min. The resulting mixture was transferred to a 23 mL Teflon-lined stainless-steel container, sealed and placed in an oven maintained at 145°C for an hour. The autoclave was then allowed to cool down naturally. The final, black-colored solid product was centrifuged and cleaned several times with an ethanol/deionized water mixture. The resultant product was then dried at 60°C in a vacuum oven.

### 2.3 Electrode preparation

Before conducting our electrochemical and electrocatalytic tests, we have prepared nickel telluride electrodes on carbon cloth substrate. After mixing 2.0 mg of catalyst powder with 300.0 µL of isopropyl alcohol (IPA) and Nafion solution (50 µL of 1% Nafion solution in 150 µL of 50% IPA in water) for 30 min, a homogenous catalyst ink was obtained. 100 μL of the catalyst-Nafion dispersion was drop-casted on carbon cloth electrode inside a confined area (geometric area of 0.283 cm^-2^) and a total catalyst loading of ∼0.67 mg was obtained. The drop-casted electrode composite was dried at room temperature before being heated in an oven at 60°C for 30 min.

## 3 Characterization

### 3.1 Materials characterization

The hydrothermally synthesized nickel telluride was characterized by powder X-ray diffraction (pxrd) using a Philips X-Pert X-ray diffractometer (PANalytical, Almelo, Netherlands) with CuKα (1.5418 Å) radiation. Scanning electron microscopy (SEM) images of the as-synthesized nickel tellurides powder were obtained using Helios Hydra field-emission microscope. The Raman spectra of all samples were collected with LabRam ARAMIS (HORIBA Jobin-Yvon Raman spectrometer equipped with a CCD detector) The nickel tellurides were analyzed by X-ray photoelectron spectroscopy XPS with a KRATOS AXIS 165 X-ray photoelectron spectrometer (Kratos Analytical Limited, Manchester, United Kingdom) equipped with a mono-chromatic Al X-ray source. The C 1s signal at 284.5 eV was utilized as a reference to adjust all the XPS binding energies. All XPS spectra were obtained from the unaltered catalyst surface without sputtering. Bandgap determination for the synthesized samples was conducted using Diffused Reflectance Spectroscopy (DRS) on an Agilent Cary 5000 UV–Vis-NIR spectrophotometer. The analysis included a Praying Mantis attachment, with BaSO_4_ serving as a white background reference.

### 3.2 Electrochemical measurements

All electrochemical measurements were conducted with an IviumStat potentiostat. The electrochemical experiments were performed in three-electrode cell system with a graphite rod as the counter electrode, Saturated Calomel Electrode (SCE) as the reference electrode and catalyst loaded carbon cloth as working electrode. All of the potentials measured throughout this investigation were converted to the reversible hydrogen electrode (RHE) scale using Eq. 1.

$$E\left(\text{RHE}\right)=E\left(\text{SCE}\right)+E{}^{\circ}\left(\text{SCE}\right)+0.059\text{ pH}$$ (1)

After acquiring linear sweep voltammetry (LSV) curves at a scan rate of 5 mV s^-1^, the electrochemical workstation automatically corrected them *via* iR compensation. Electrochemical impedance spectroscopy (EIS) was used to examine the charge transfer resistance from 0.01 Hz to 100 kHz at a voltage of 1.50 V vs RHE. One Sun illumination was used to examine the transient photocurrent response of the as synthesized samples,.

The Tafel slope, obtained by fitting polarization data to the Tafel equation, is a vital parameter for assessing the OER activity. The relationship between the overpotential and the current density j) is expressed through the Tafel equation, as illustrated in Eq. 2:

$$\eta =a+\left(\frac{2.3\text{RT}}{\alpha \text{nF}}\right)\log\;\left(j\right)$$ (2)

where n is the number of electrons involved in the reaction, α is the transfer coefficient, and F is the Faraday constant. The Tafel slope is given by 2.3RT/αnF.

The Electrochemically active surface area (ECSA) was calculated by employing double-layer capacitance (C_DL_) as per Eq. 3:

$$ECSA=C_{DL}/C_{S}$$ (3)

Where C_DL_ is the double layer capacitance and C_S_ is the specific capacitance. Similar to previously reported metal selenide-based catalysts, Cs = 0.04 mF cm^–2^ was employed to analyze ECSA (Lee et al., 2012; Ahsan et al., 2020; Kale et al., 2020; Oh et al., 2020). The C_DL_ was calculated by averaging the absolute values of cathodic and anodic slopes.

### 3.3 Photoelectrochemical measurements

The photocurrent–photovoltage curves were obtained using LSV with the scan rate set at 5 mV s^–1^ and the stirring speed set at 200 rpm. A 100-W ozone-free xenon Oriel LCS-100 lamp with an AM 1.5 filter served as the primary source of simulated solar illumination.

The Mott-Schottky (MS) analysis was conducted to determine the positions of the conduction band and valence band edges in NiTe. The Mott-Schottky plots for NiTe-electrode were obtained at a frequency of 100 Hz, and the band-edge potentials were estimated using Eq. 4 (Wu et al., 2012; Singh et al., 2020).

$$\frac{1}{C^{2}}=\left[\frac{2}{\in \in_{0}\text{eN}}\right]\left[E-E_{\text{fb}}-\frac{\text{kT}}{E}\right]$$ (4)

Here, E represents the applied potential, and E_fb_ denotes the flat-band potential. C stands for the charge capacitance, ɛ is the dielectric constant of the semiconductor, ɛ_0_ is the permittivity of vacuum, k is the Boltzmann constant, e signifies the electron charge (with +e and −e for electrons and holes, respectively), and T denotes the temperature.

### 3.4 DFT calculation

The Vienna *ab initio* Simulation Program (VASP) ver. 5.4.4 was used to perform DFT calculations using the generalized gradient approximation (GGA) that is described by the Perdew–Burke–Ernzerhof (PBE) exchange-correlation functional (Perdew et al., 1996). For all calculations, the slab containing a 3 × 3 supercell of NiTe was created. A kinetic energy cutoff of 520 eV was used and integration was carried out over the Brillouin zone using a 9 × 9 × 1 Monkhorst-Point k-point mesh and the Gaussian smearing method with a sigma value of 0.05 eV. To prevent any erroneous contact, a vacuum height of ∼10 Å along the vertical direction was used to position the slab separated from its periodic images.

## 4 Results and discussion

**FIGURE 1:**
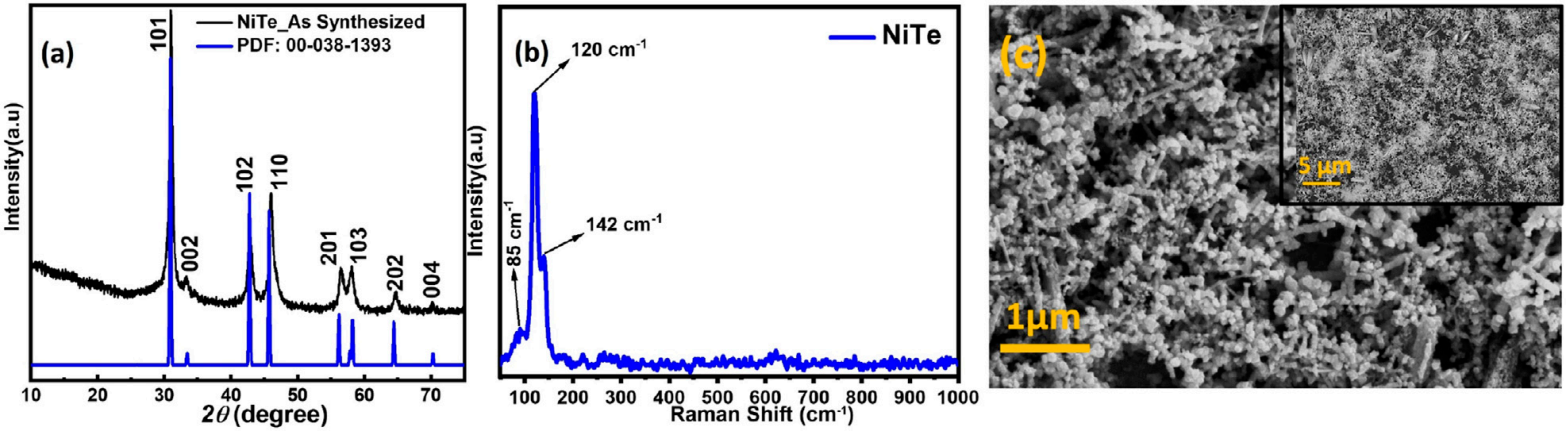
The **(A)** PXRD pattern, **(B)** Raman spectra and **(C)** SEM image of as-synthesized NiTe powder.

Figure 1A shows the typical PXRD pattern of the as-synthesized powder which confirmed formation of NiTe. A comparison of the experimental pattern with the standard diffraction file of hexagonal NiTe, (JCPDS: 38–1393) revealed a perfect match and the diffraction peaks observed at 31.54°, 43.41°, 46.52°, and 58.61° could be attributed to the (101), (102), (110), and (103) crystallographic planes, respectively. In the XRD pattern, no other diffraction peak is seen, indicating that no other crystalline product has formed. It must be noted that such high purity NiTe was obtained through the simple one-pot hydrothermal synthesis within 1 h of reaction time. Traditional methods for synthesizing metal chalcogenides typically take days or even weeks to complete, while this new approach only takes an hour. Such short reaction time, moderate temperature, along with high phase purity of the obtained NiTe product makes this an excellent approach for scalable and economically feasible catalyst development.

**FIGURE 2:**
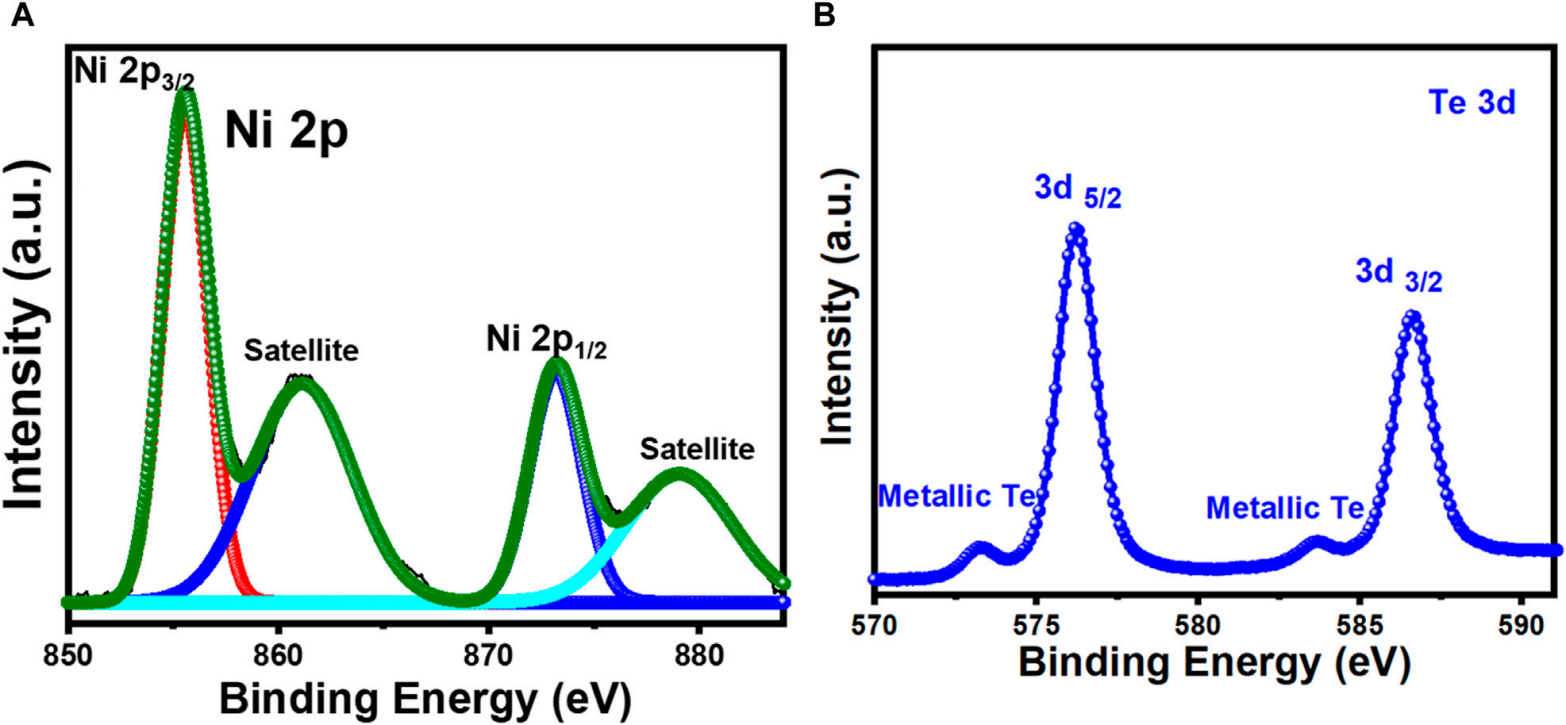
High resolution XPS spectra of **(A)** Ni 2p and **(B)**Te 3d.

Raman spectra was used to further confirm the composition of these nanostructures. As demonstrated in Figure 1B, Raman peaks were observed at 124 and 143 cm^-1^ which can be attributed to the NiTe. The Raman peaks that are positioned between 100 and 150 cm^-1^ are correlated with the telluride (Guo et al., 2022). Shi et al. observed in their study that the Raman spectra of NiTe showed distinct peaks at 85 cm^-1^, which represents the Ni-Te bond (Shi et al., 2020; Deng et al., 2022). Figure 1C depicts the surface morphology of NiTe nanostructures. The NiTe has elongated nanostructure with diameters of approximately 80–100 nm, as shown by the low magnification FESEM image (Figure 1C). Some of the NiTe elongated nanostructure have a propensity to cluster together. The nanostructured ensemble exhibited by the NiTe provides larger surface area, ensuring a greater number of exposed electrocatalytic active sites. This morphological feature maximizes the potential for efficient access of electrolytes during electrochemical reactions, thereby enhancing the overall OER activity. The elemental composition and their oxidation states were further investigated through XPS measurements. Ni XPS spectra showed two spin-orbit doublets as well as two shakeup satellites as shown in Figure 2A. Peaks at 853.6 and 870.8 eV correspond to Ni^2+^ in NiTe. Furthermore, the peaks at approximately 861.7 and 878.9 eV have been assigned to the respective shakeup satellites (Wang and Zhang, 2018). The XPS peaks at 586.6 and 576.7 eV are attributed to Te 3d3/2 and Te 3d5/2 in NiTe, respectively, as shown in Figure 2B (Wang and Zhang, 2018). The appearance of two additional, minor peaks at 573.4 and 583.8 eV are indicative of zero-valent Te. It should be noted here that presence of such trace amount of Te does not negatively impact the photoelectrocatalytic response of the NiTe layer significantly as shown in the following sections.

**FIGURE 3:**
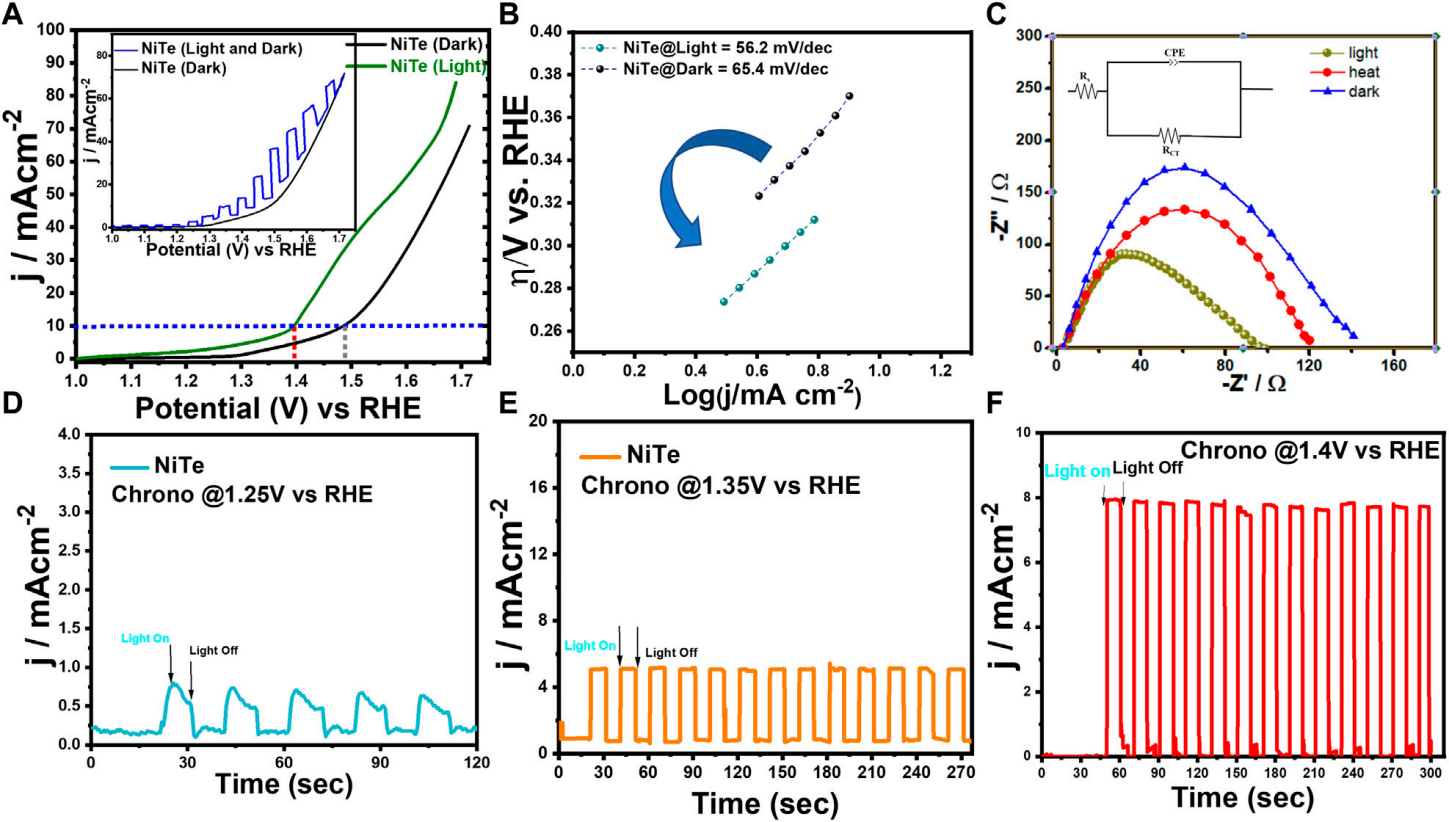
**(A)** The LSV curves of NiTe deposited on carbon cloth on measured in the dark and under light illumination. Inset shows the current response in dark and chopped illumination. **(B)** The Tafel plots derived from the LSV measurements from dark and under illumination. **(C)** The EIS curves of NiTe measured under light illumination, dark and heating up to 36°C. **(D–F)** Photocurrents response of as deposited NiTe films at potentials of 1.25, 1.35 and 1.4 V *versus* RHE in 1 m KOH solution under illumination.

Detailed electrochemical measurements were performed in a 3-electrode set-up as described above to evaluate the electrocatalytic performance of NiTe for OER under dark and illuminated conditions. The electrochemical measurements were performed in an N_2_-saturated 1.0 M KOH electrolyte. Supplementary Figure S1 illustrates the Electrochemically Active Surface Area (ECSA) of the synthesized NiTe catalyst. The ECSA was determined for the NiTe electrode by utilizing double-layer capacitance in the non-faradaic region, extracted from cyclic voltammetry (CV) plots, as depicted in the inset of Supplementary Figure S1. Capacitive currents at 0.18 V vs SCE were used from each CV at different scan rates in 1 M KOH to calculate the ECSA. The as-prepared electrode exhibited an estimated ECSA of 36.51 cm^2^. A larger ECSA correlates with better charge transfer capabilities and improved exposure of active sites leading to enhanced OER efficiency. All samples were analyzed using iR-corrected linear sweep voltammetry LSV curves. Figure 3A shows that NiTe nanostructures display intrinsic OER activity in the absence of light irradiation, with overpotentials as low as 261 mV to produce current densities of 10 mA cm^-2^. Interestingly the OER activity of the NiTe nanostructures shows a significant enhancement under illumination with 1.5 AM simulated solar light source. After being exposed to solar light, the overpotential of NiTe nanostructures clearly shifted to 165 mV at 10 mA cm^-2^ current density, which is significantly less than that acquired in the absence of light. Inset of Figure 3A shows the comparison of the LSV curves of the NiTe catalyst in dark and under chopped illumination. The reduced overpotentials suggest that the OER activity of the NiTe nanostructures in a well-designed solar-intensified electrocatalytic system might be significantly increased by the light illumination. The incident light on the electrode increases the catalyst activity by creating a more favorable environment for the OER. It also increases the amount of charge transfer between the catalyst-electrode surface and the electrolyte, thus reducing the overpotential of the reaction.

It was observed that continuous illumination while measuring LSV, led to slight increase of the electrolyte temperature to 36°C. To confirm the enhanced OER activity as shown in Figure 3 is indeed due to light illumination and not effect of increased temperature, we have compared the OER activities of the same NiTe-electrode under solar illumination with that where the electrolyte was heated to 36°C without any illumination as shown in Supplementary Figure S2. The comparison LSV plot clearly shows that even though the system was heated up to 36°C by external heat supply, the LSV curve of the NiTe exhibits minimal change in the absence of light illumination, showing that the OER activity of the NiTe cannot be obviously increased by this slight increase in temperature of the electrolyte. In contrast, the OER activity clearly improved in presence of light illumination of NiTe, as demonstrated in Figure 3A further confirming the solar-intensified OER activity of NiTe. The observed enhancement in OER activity under light illumination, surpassing the impact of uniform system heating, can be attributed to the distinct mechanisms involved in photothermal conversion. Photothermal effects induced by light lead to localized temperature increases specifically at the electrode surface where the electrocatalytic reactions take place. This localized temperature enhancement influences the reaction kinetics and facilitates more efficient activation of catalytic sites, thereby improving the overall electrocatalytic performance. Unlike uniform heating of the entire system, which may not effectively concentrate thermal energy at the reaction sites, photothermal conversion optimizes the distribution of heat precisely where it is needed for catalysis. This targeted thermal enhancement, coupled with the inherent properties of the material, results in a more pronounced improvement in OER activity. Consequently, these findings underscore the strategic advantage of harnessing photothermal effects to enhance electrocatalytic performance, providing valuable insights for optimizing oxygen evolution reactions in various applications.

The Tafel slope deduced from LSV is an important parameter used to describe the kinetics of the electrochemical process. It is defined as the negative of the slope of the logarithmic current as a function of overpotential, or the rate at which the current density increases with increasing overpotential. The Tafel slope is an important parameter for understanding the electrokinetic behavior of catalysts used for the OER and for predicting the performance of fuel cell systems. The Tafel slope is also influenced by the nature of the catalyst, the electrolyte, and other factors. The Tafel slope for NiTe under one Sun illumination was estimated to be only 56.2 mV dec^−1^, which is lower than the Tafel slope for NiTe in the dark (65.4 mV dec^−1^). This signifies that the favorable reaction kinetics of NiTe in electrocatalytic oxidation can be clearly boosted under solar illumination (Zhang et al., 2020).

To investigate the OER kinetics in the presence and absence of illumination, electrochemical impedance spectroscopy (EIS) studies were performed. Figure 3C shows a typical Nyquist plot for NiTe-modified electrode, which reveals a clear decreasing trend in charge transfer resistance (R_ct_) for the as synthesized electrode under light illumination. The NiTe sample, which demonstrates the smallest R_ct_ (74 Ω) in presence of solar light, reveals that it has the fastest electrocatalytic reaction kinetics under all conditions. Additionally, Supplementary Table S1 presents the equivalent circuit parameters derived from fitting the EIS experimental data for the NiTe sample under conditions of light, heat, and darkness. The ameliorated electrical conductivity during the OER process can be largely attributed to the solar energy absorption, as evidenced by the lower charge transfer resistance. This improvement is the combined effect of large number of carrier generation through illumination and faster transport induced by the localized increase in temperature produced by the solar-induced thermal effect. These findings suggest that solar energy can effectively increase the kinetics and thermodynamics of OER in NiTe-based electrodes.

Figures 3D–F depicts the j^-t^ curve used to investigate the solar-induced current density of NiTe at applied potentials of 1.25, 1.35, and 1.4 V vs RHE. In addition to the photothermal effect on OER performance discussed above, the photoelectric effect caused by light irradiation may also play a crucial role in affecting OER performance. Chronoamperometric j^-t^ measurements were performed at various applied potentials to record the transient photocurrent response of the NiTe electrode under chopped illumination, with an aim to ascertain whether or not such an effect also ameliorates the OER performance. When the light was turned on, there was an increase in the current density from 0.06 to 0.64, 5.6, and 7.86 mA cm^-2^ at an applied potential of 1.25, 1.35, and 1.4 V (vs RHE) respectively, while switching off the light reduced the current densities close to their respective base values. This intermittent on-off experiments also confirmed that the enhancement of current density is due to increase in the charge carriers formed in NiTe under illumination. The density of charge carriers in the electrocatalyst are enhanced through the formation of excitons by the absorption of photons with sufficient energy. The presence of additional charge carriers can increase the rate of reaction on the catalyst surface leading to enhanced OER activity.

**FIGURE 4:**
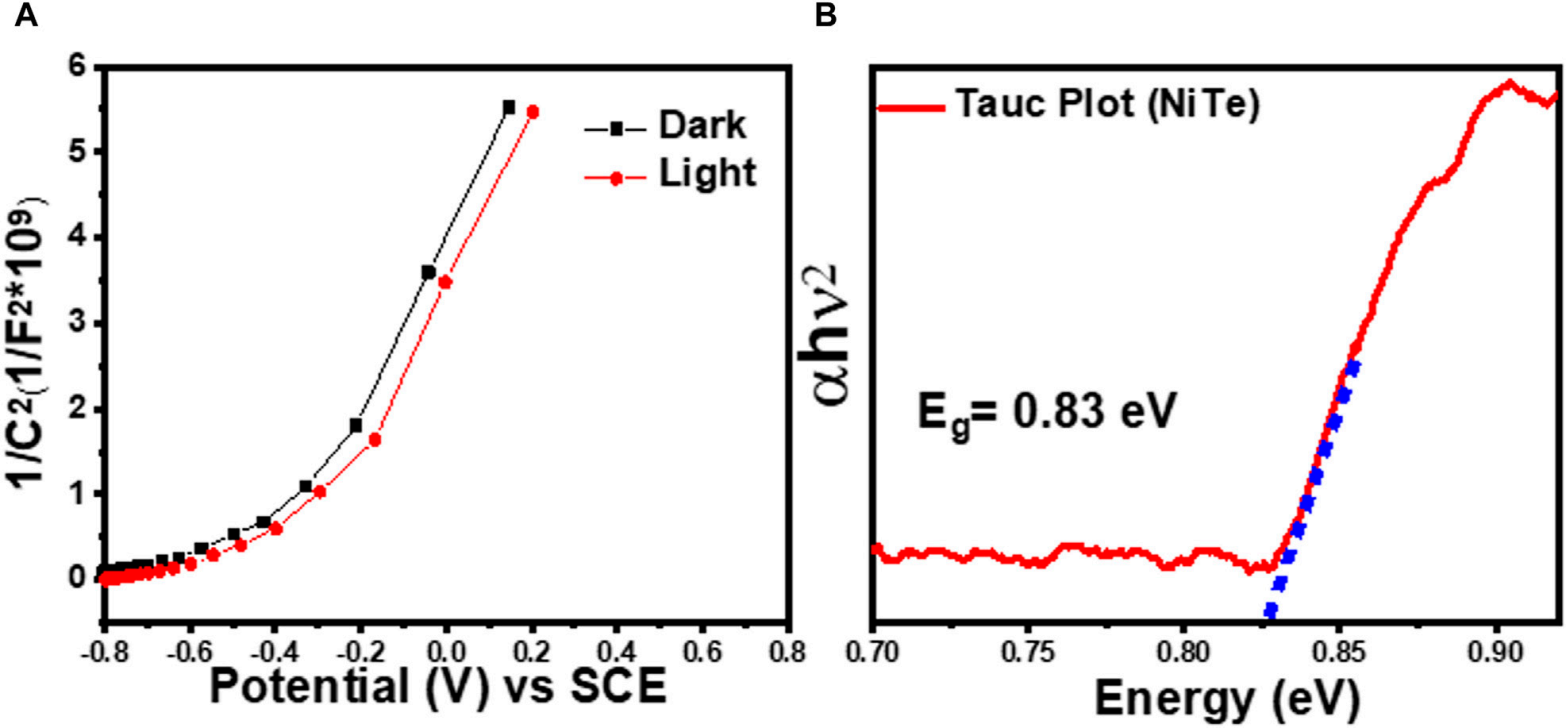
**(A)** Mott-Schottky plots at 100 Hz of NiTe in dark and light. **(B)** Plots of (αhv)^2^ vs photon energy (hν) obtained using UV−Vis diffuse reflectance spectra of NiTe.

Figure 4A shows the Mott–Schottky plots of the prepared NiTe electrode. A Mott-Schottky plot, correlating 1/C^2^ with the applied potential, reveals crucial insights into the flat band potential (V_FB_) and the electrode’s nature. As depicted in Figure 4A, the negative V_FB_ of NiTe suggests efficient photogenerated charge carrier separation at the interface. Furthermore, the positive slope in the plot indicates the n-type nature of the prepared electrode (Singh et al., 2020). The doping density can be estimated from the slope of the linear fit of the Mott-Schottky plots using the equation shown in Figure 4. However, for such analysis, a more accurate estimation of the dielectric constant of the semiconductor layer is needed. Linear fit of the Mott-Schottky plots, on the other hand, revealed the flat-band potentials of NiTe under dark and light conditions to be −0.42 and −0.39 V vs Ag/AgCl, respectively. The shift towards a more negative flat-band potential under light conditions, as observed in the Mott-Schottky curves for NiTe, is indicative of a phenomenon known as the photogenerated charge carrier separation. In the presence of light, photon absorption leads to the generation of electron-hole pairs within the material. The resulting photogenerated charge carriers, particularly electrons, influence the electrostatic interactions at the semiconductor-electrolyte interface. When light is incident on NiTe, the absorption of photons energizes electrons, elevating them to higher energy states. This promotes efficient charge separation, causing a surplus of electrons near the surface. As a result, the flat-band potential becomes more negative compared to the dark condition. The negative shift reflects the enhanced separation and accumulation of photogenerated electrons at the semiconductor-electrolyte interface during illumination. This phenomenon is crucial for applications like photoelectrochemical cells, where efficient charge separation is essential for harnessing light energy (Zhuang et al., 2018; Sajeev et al., 2022).

The conduction band of the n-type semiconductor is positioned in close proximity to the flat bandgap. Consequently, the conduction band potential (E_CB_) for pure NiTe is also determined to be −0.39 V, aligning with the semiconductor’s intrinsic characteristics. In Figure 4B, analysis of the UV-vis diffuse reflectance spectra further reveals a bandgap of 0.83 V for NiTe. Leveraging the relationship between E_CB_ and bandgap (E_BG_), the valence band potential (E_VB_) is calculated for pure NiTe, resulting in a value of 0.44 V. This methodology provides insights into the electronic structure and band alignment of NiTe.

**FIGURE 5:**
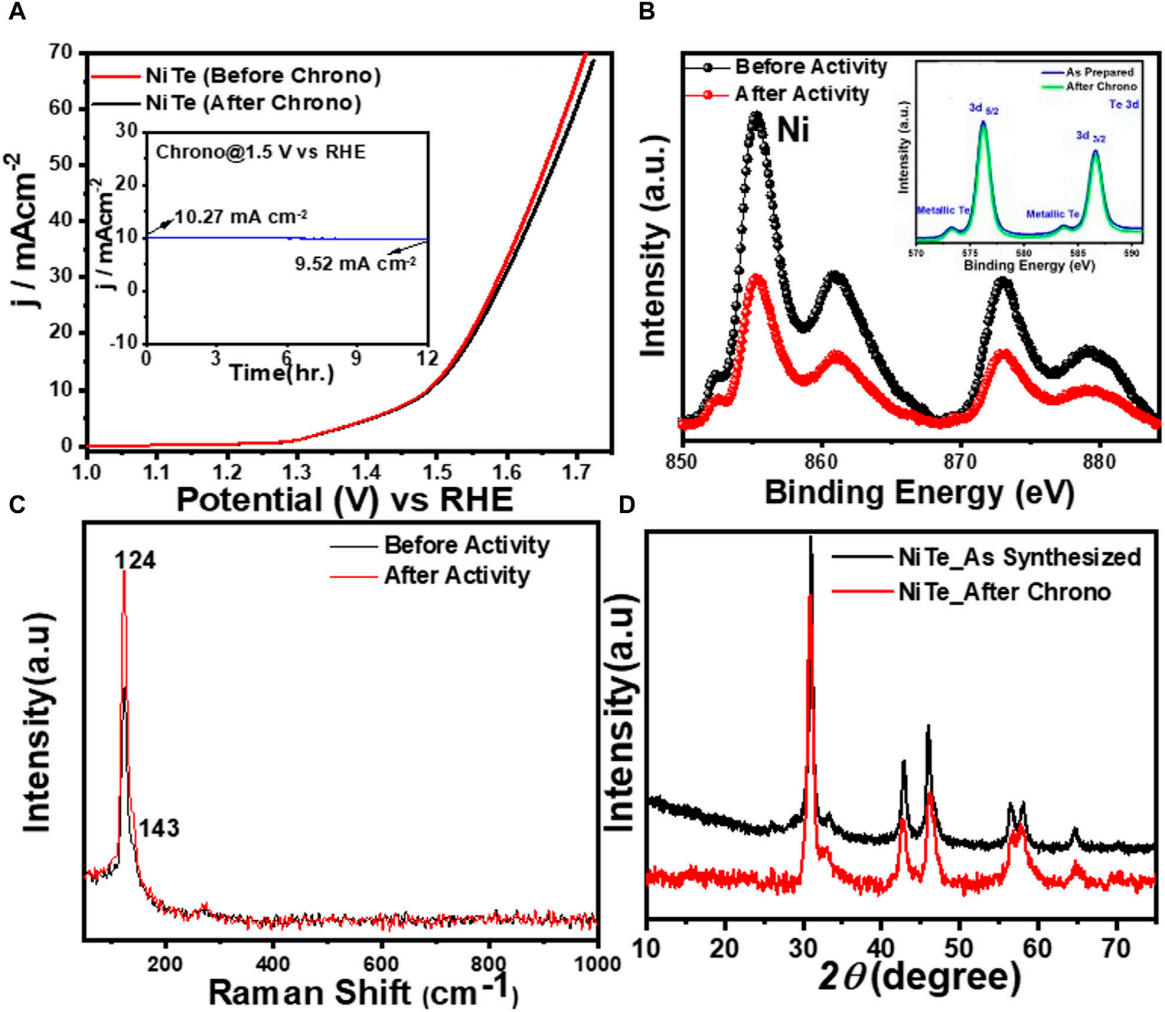
**(A)** LSV of NiTe before and after 12 h of chronoamperometry. Inset shows the chronoamperometry test at applied potential of 1.5 V vs RHE. **(B)** High resolution XPS spectra of Ni 2p, and Te 3d (inset) before and after chronoamperometry. **(C)** High resolution XPS spectra and **(D)** PXRD pattern of NiTe before and after chronoamperometry.

In addition, the stability test for the NiTe catalyst was carried out in alkaline conditions by chronoamperometry at an applied constant potential of 1.5 V vs RHE. According to Figure 5A, the NiTe catalyst exhibited remarkable stability over a substantial period of time. As demonstrated in Figure 5A, even after 12 h of long-term OER, the NiTe sample exhibited no change in LSV plots while the chronoamperometry showed no deterioration of the current density (Figure 5A inset). The composition and morphology of the electrocatalyst after prolonged activity in the presence and absence of illumination was investigated using XPS, Raman spectroscopy, and powder X-ray diffraction. As demonstrated in Figure 5B, the XPS data of the NiTe exhibited no change in XPS peak positions of either Ni or Te, indicating the compositional stability of the catalyst composite. Similarly, the Raman spectra of the NiTe catalysts (Figure 5C) showed no shift in peaks and no new peaks after 12 h of OER, indicating that no structural changes occurred in the NiTe catalytic composite. After 12 h of OER in alkaline media, the crystallinity and phase purity of the NiTe phases were not altered, as seen by the PXRD patterns in Figure 5D. Consequently, based on the results of the PXRD, XPS, and Raman spectra, it can be concluded that there was no degradation or bulk transformation of the NiTe catalyst after sustained OER under applied anodic potential. These analyses provide confirmation of the excellent stability of the NiTe catalyst, without any performance degradation.

**FIGURE 6:**
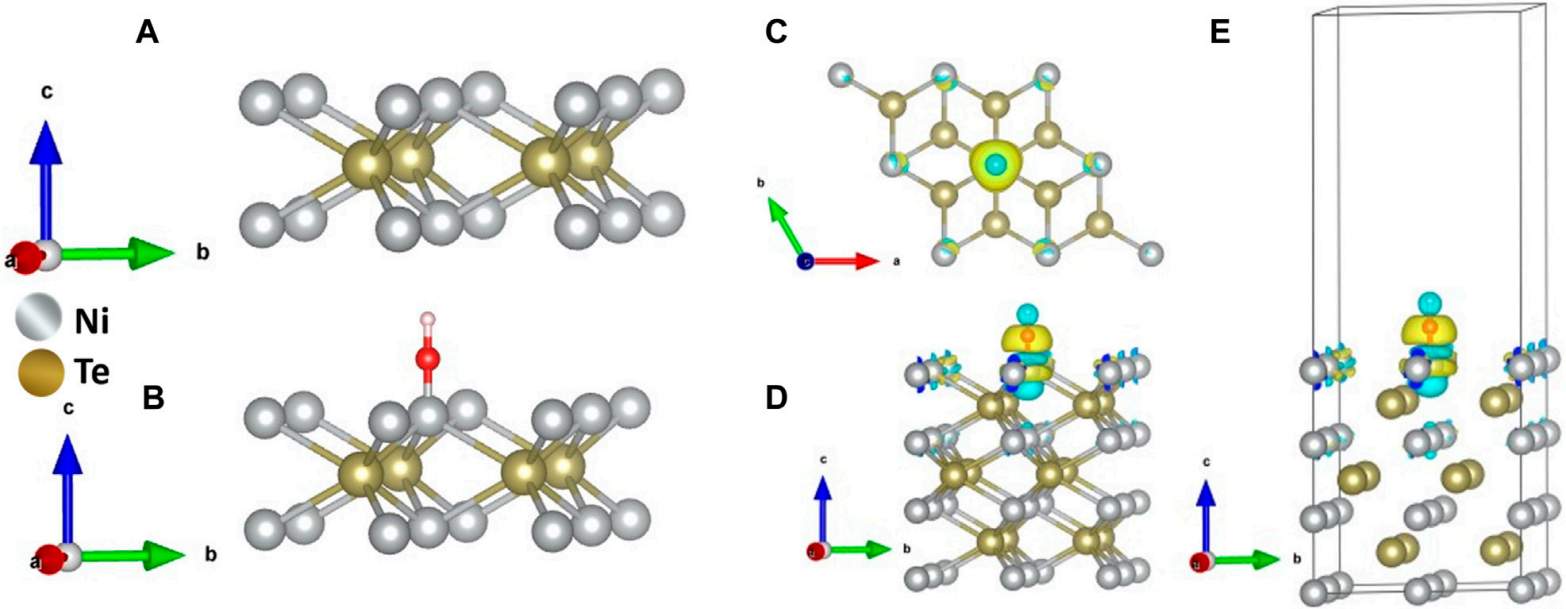
Schematic representation of the top layer of the NiTe {001} slab **(A)** without and **(B)** with OH adsorption. **(C)** A charge density map **(D)** top view, and **(E)** side view, when a OH molecule adsorbed to the Ni catalytic site.

Density functional theory (DFT) calculations were used to further analyze activation of the catalyst surface through -OH adsorption, which has been considered as one of the primary steps for OER catalytic activity. The (001) surface of NiTe was used for DFT calculations, where OH was adsorbed on the catalytically active Ni site. The optimal top layer structures of the NiTe (001) structure without and with OH adsorption are shown in Supplementary Figure S3, Figures 6A, B. Figures 6C–E illustrate the differential charge densities after adsorption of OH on Ni sites of NiTe. Furthermore, the interaction between OH and NiTe surfaces leads to an intriguing phenomenon of redistribution of charges. This redistribution occurs due to the electronic hybridization that transpires between the orbitals of the adsorbate (OH) and those of the adsorbent (NiTe). Such charge density redistribution is a common occurrence accompanying the process of adsorption. It is an important feature to consider when examining the effects of chemical interactions at surfaces, as it can have significant implications for the material’s electronic and chemical properties. In our current study, we have employed a charge analysis technique to delve into the changes in charge density. This approach enables us to gain a deeper understanding of the adsorption mechanism at play. It allows us to pinpoint where electrons are gained or lost and provides valuable insights into the interactions between the adsorbate and adsorbent. This understanding is crucial for unraveling the underlying mechanisms and behaviors of chemical reactions and surface interactions. According to the calculated charge density differences analysis (Figures 6C–E), Te atom was accustomed to donating electrons to Ni center. This characteristic could lead to a strong coupling between Ni atoms and hydroxyl species.

This interaction is important because the catalytic activity in OER involves catalyst site activation through the adsorption of OH. When hydroxyl species, which tend to attract electrons (electron-drawing), are added, there’s a noticeable charge transfer occurring at the Ni-OH interface. Specifically, electron density moves from the Ni center to the oxygen O) atom of the hydroxyl group. This charge transfer is significant because it affects how electrons are distributed in the catalyst, influencing its catalytic behavior. The depleted electron density on the catalytic site reduces the overpotential for further charge transfer thereby enhancing the electro-oxidation reaction. Furthermore, the adsorption energies of OH on Ni sites were estimated on (001) facets of NiTe. Adsorption energy is a critical factor in catalysis as it influences how strongly or weakly the reactants interact with the catalyst surface. Understanding these energies can help in tuning the catalyst for optimal performance. Supplementary Figure S4 in Supplementary Material shows the structural models and energies of OH adsorption. The OH-binding energy was calculated to be–1.64 eV at the Ni -site. Supplementary Figure S4 provides insight into the Total Density of States (TDOS) concerning the Ni 3d orbital before and after the attachment of the OH group. This data reveals a notable shift in the occupied spin-up Ni states towards lower negative energy levels upon the binding of OH-. This shift can be attributed to the weakening of the Ni-Te interactions as a consequence of OH attachment.

## 5 Conclusion

Nickel telluride, a non-precious transition metal based chalcogenide, has been identified as a highly efficient electrocatalyst for solar-enhanced water splitting leading to very low overpotential for oxygen evolution reaction. Moreover, the NiTe could be synthesized through a 1-h hydrothermal reaction at a remarkably low temperature of just 145°C. The synthesized NiTe elongated nanostructure exhibited exceptional OER activity, evident from the low overpotential values of 261 mV in the dark and 165 mV under simulated solar illumination. DFT studies were performed to investigate the electronic interactions within NiTe, particularly focusing on how electron transfer and intermediate hydroxyl adsorption characteristics on the catalyst site can influence its efficiency as a catalyst in OER processes. Such understanding of the intrinsic material properties towards enhance electrochemical activity is fundamental in designing and optimizing materials for energy conversion applications. Moreover, the demonstrated durability of the material positions it as a highly prospective OER electrocatalyst for various applications, offering significant implications for the advancement of electrochemical technologies. The NiTe catalyst, with its combination of high activity and remarkable stability, makes it competitive with the noble metal catalysts RuO_2_ and IrO_2_. This report not only demonstrates a significant advancement in the field of OER electrocatalysts, but also opens up new possibilities for the rapid and controlled fabrication of functional telluride nanostructures.

## Data Availability

The raw data supporting the conclusion of this article will be made available by the authors, without undue reservation.
