# Supplementary material for: Solar enhanced oxygen evolution reaction with transition metal telluride
Source: Front Chem. 2024 Apr 26;12:1381144. doi: 10.3389/fchem.2024.1381144 (PMC11082350; doi:10.3389/fchem.2024.1381144)
Supplement: Supplementary file 1 [file DataSheet1.PDF]

Solar Enhanced Oxygen Evolution Reaction with Transition metal telluride

Harish Singh[a], Taishi Higuchi-Roos[ac], Fabrice Roncoroni[b], David Prendergast[b], and Manashi Nath[a]*

[a] Department of Chemistry, Missouri University of Science and Technology, Rolla, MO 65409.

[b] Joint Center for Energy Storage Research, the Molecular Foundry, Lawrence Berkeley National Laboratory, Berkeley, California 94720, United States

[c] Department of Chemical and Biochemical Engineering, Missouri University of Science and Technology, Rolla, MO 65409, USA

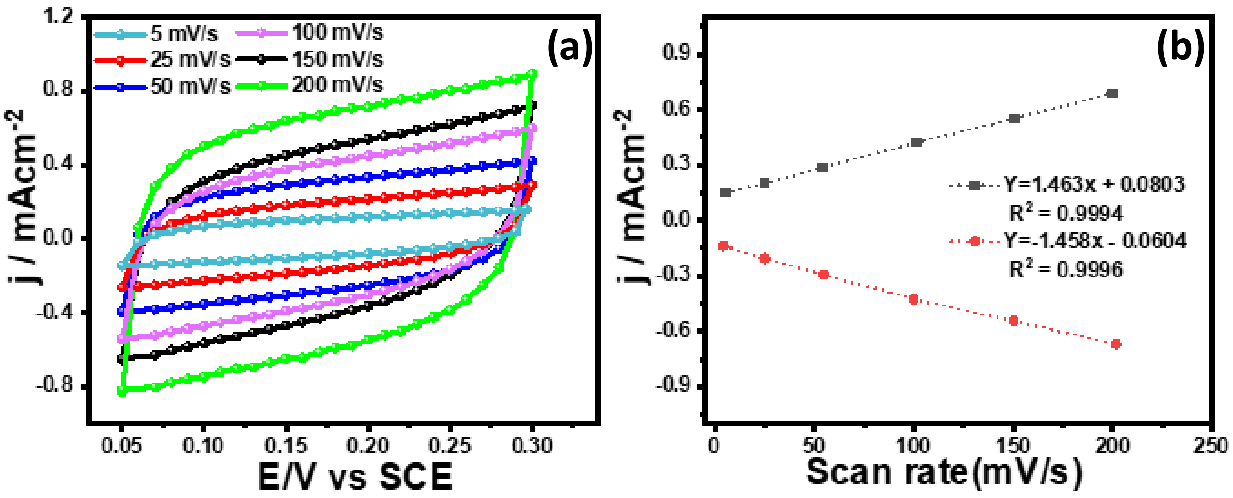

Figure S1. (a) Cyclic voltammograms measured at different scan rates for NiTe. (b) Plots of anodic and cathodic currents measured as a function of different scan rates for NiTe.

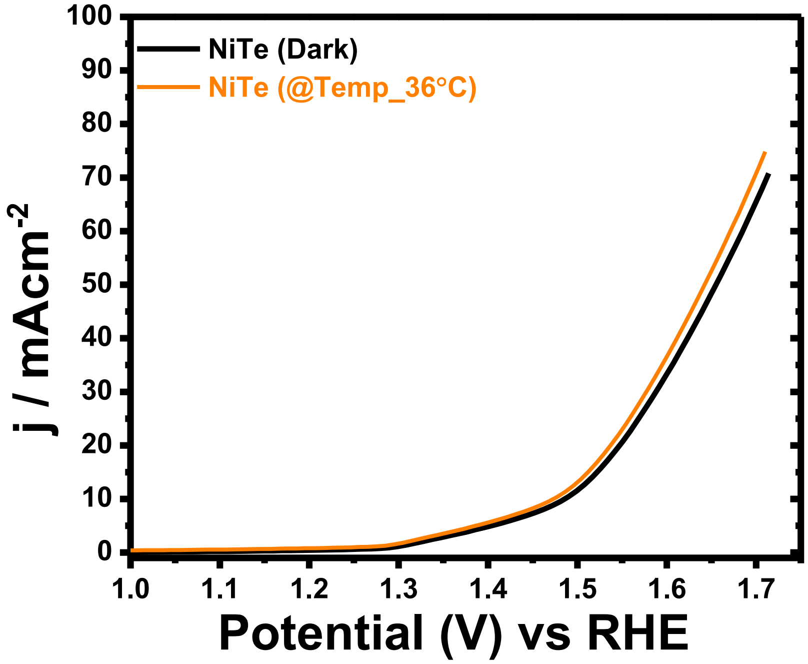

Figure S2. LSV of NiTe in dark and under illumination at 36°C temperature.

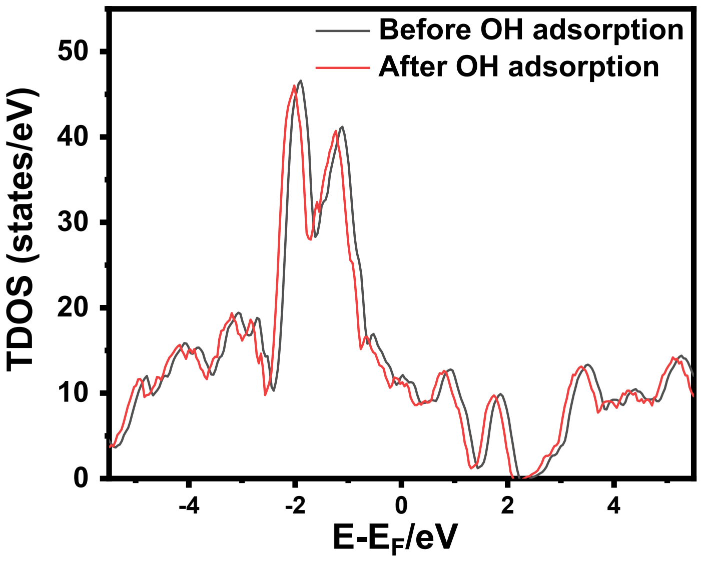

Figure S3 The total density of states of OH adsorbed on NiTe

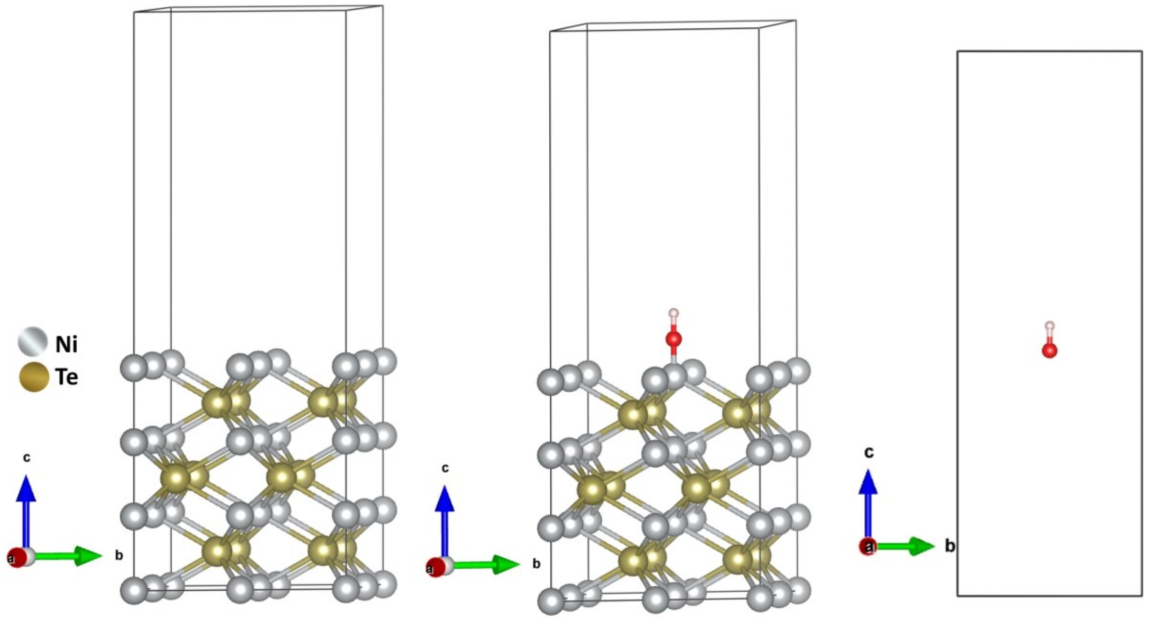

Figure S4. Relaxed lattice structures of (a) NiTe, (b) NiTe with OH adsorption, and (c) NiTe without OH adsorption on active Ni sites on (001) free surfaces.

Table 1. Equivalent Circuit Parameters Obtained from Fitting of EIS Experimental Data

Parameter	Light	Heat	Dark
**R_s/Ω**	8.43	9.62	8.21
**R_{ct}/Ω**	74	112	134
**$CPE_{catalyst}$/nF**	0.82	1.21	0.96
